# Identifying and managing refractory migraine: barriers and opportunities?

**DOI:** 10.1186/s10194-019-1040-x

**Published:** 2019-08-23

**Authors:** Linda D’Antona, Manjit Matharu

**Affiliations:** Headache and Facial Pain Group, UCL Queen Square Institute of Neurology and The National Hospital for Neurology and Neurosurgery, Queen Square, London, WC1N 3BG UK

**Keywords:** Migraine, Refractory, Medication overuse, Preventive, Abortive, Disability, Comorbidities

## Abstract

The term refractory migraine has been used to describe persistent headache that is difficult to treat or fails to respond to standard and/or aggressive treatments. This subgroup of migraine patients are generally highly disabled and experience impaired quality of life, despite optimal treatments. Several definitions and criteria for refractory migraine have been published, but as yet, an accepted or established definition is not available. This article reviews the published criteria and proposes a new set of criteria. The epidemiology, pathophysiology and management options are also reviewed.

## Introduction

Migraine is a chronic paroxysmal neurological disorder characterised by attacks of moderate or severe headache and reversible neurological and systemic symptoms. The most characteristic symptoms associated with migraine include photophobia, phonophobia, and gastrointestinal symptoms such as nausea and vomiting [1]. The management of migraine includes identifying and excluding secondary headache types, addressing comorbid factors, and optimizing both pharmacological management and behavioural treatments. Although much progress has been made in recent years in the management of migraine, there remains a group of patients who continue to experience disabling headache despite optimal treatment. These patients remain “refractory” or “intractable” to standard treatment. However, universally accepted definitions of “refractory” or “intractable” are not available.

## Historical perspective

The term “refractory migraine” was first used by Reisman in 1952 when he reported the use of suppositories of ergot-alkaloids to treat migraine [2]. However, little attention was subsequently paid to this term until just over a decade ago. Over the last decade there have been several attempts to define refractory migraine albeit that there is still a lack of consensus on this issue.

Goadsby et al proposed a definition of intractable migraine and cluster headaches in 2006 [3]. It required the failure of four preventive drugs applicable to the type of headache being treated. Acute treatments and degree of disability were not included in these criteria. In 2008, the Refractory Headache Special Interest Section (RHSIS) of the American Headache Society (AHS) definition of refractory migraine were published [4]. These criteria required only failure of two classes of preventive treatments. In addition, patients needed to fail 3 classes of acute treatments. Medication overuse and degree of disability were included as modifiers. Silberstein et al proposed a definition for pharmacologically intractable headache in 2010 [5]. They build upon the AHS criteria, proposing a graded classification scheme for intractability to acute and preventive treatments as well as rating of headache-related disability. The European Headache Federation (EHF) provided a consensus statement on the definition of chronic migraine (CM) in 2014 [6]. These criteria are restricted to CM and require the failure of three classes of preventive treatments. They require adequate treatment of psychiatric or other comorbidities by a multidisciplinary team, if available, but acute treatments and degree of disability were not included in these criteria.

An overview of these proposals reveals that there is a lack of consensus on the definition of refractory migraine as well as the factors included in their operational criteria (see Table 1).

**Table 1:** Overview of the published proposal for refractory or intractable migraine

	Goadsby et al. (2006)	Schulman et al. (2008)	Silberstein et al. (2010)	Martelletti et al. (2014)
Headache Diagnosis				
Diagnostic criteria	Not stated	ICHD diagnostic criteria	ICHD diagnostic criteria	ICHD diagnostic criteria
Episodic or Chronic Migraine	Not stated	Both included	Inclusion of both episodic and chronic migraine not explicitly stated but implicitly included as criteria pertain to all primary headaches	Limited to chronic migraine
Medication overuse	Not stated	With or without medication overuse (as defined by ICHD criteria) included as modifier	Not stated	No medication overuse
Acute Treatments				
Inclusion in operational criteria	Criteria limited to preventive treatments only	Included in refractoriness criteria	Included in refractoriness criteria	Criteria limited to preventive treatments only
Number of acute treatments failed	Not applicable	Both of the following 2 classes	Grading system proposed: Mild: failed class1 Moderate: failed classes 1–3 Severe: failed classes 1–4	Not applicable
Acute classes	Not applicable	1. Both a triptan and DHE intranasal or injectable formulation 2. Either nonsteroidal anti-inflammatory drugs or combination analgesics	1. Non-specific acute treatments (eg, NSAIDs, combination analgesics) 2. Triptans or ergot derivatives 3. Oral dopamine antagonists or parenteral NSAID 4. Oral or parenteral opioids or corticosteroids or parenteral dopamine antagonists	Not applicable
Preventive treatments				
Number of classes failed	4 classes (including 3 from 1 to 4)	2 of 4 classes:	Grading system proposed: Mild: failed 1 of cases 1–10 Moderate: failed 2 drugs where 1 must be from classes 1–6 Severe: failed 3 drugs where 2 must be from classes 1–6 Very severe: Above plus failed aggressive infusion or inpatient treatment and/or failure to respond to detoxification treatment in subjects with acute headache pain medication overuse	3 drugs from following classes
Preventive classes	1. Beta-blockers 2. Anticonvulsants 3. Calcium channel blockers 4. Tricyclic antidepressants 5. Other treatments with at least one positive randomised controlled trial 6. Non-steroidal anti-inflammatory drugs 7. Metabolic enhancers	1. Beta-blockers 2. Anticonvulsants 3. Tricyclics 4. Calcium channel blockers	1. Beta-blockers (shown to be effective) 2. Tricyclic antidepressants 3. Verapamil or flunarizine 4. Sodium valproate (or divalproex sodium) 5. Topiramate 6. Combination therapy that includes at least 1 drug of type 1–5; the second drug can be from any type (1-5 or 6-9). The drugs must be of different types (eg, a combination of 2 anticonvulsants is not acceptable) 7. Gabapentin 8. Other treatments with at least 1 positive placebo-controlled trial 9. Non-steroidal anti-inflammatory drugs 10. Metabolic enhancers (Vitamin B2 or CoQ10)	1.Beta blockers (propranolol up to 240 mg/d; metoprolol up to200mg; atenolol up to100mg; bisoprolol up to10mg) 2.Anticonvulsants (valproate acid up to 1,5 g/d; topiramate up to 200 mg/d) 3.Tricyclics (amitriptyline up to 150 mg/d) 4.Others (flunarizine up to 10 mg/d; candesartan 16 mg/d) 5.OnabotulinumtoxinA (155–195 U according to the PREEMPT protocol)
Headache-Related Disability	Disabled by standard scales e.g. MIDAS, HIT-6 or scale suitable in country of assessment	Included as a modifier, with patients classified as having significant disability if MIDAS > 11	Disability measured using MIDAS or HIT-6	Not included
Lifestyle factors	Not addressed	Criteria state that headaches should cause “significant interference with function or quality of life despite modification of triggers, and lifestyle factors” but no operational criteria provided	Considered by authors but excluded	Not included
Comorbidities	Not addressed	Not addressed	Considered by authors but excluded	Adequate treatment of psychiatric or other comorbidities by multidisciplinary team, if available.
Definition of failed trial	No therapeutic or unsatisfactory effect^a^ Intolerable side effects Contraindications to use	Not defined	Not defined	No therapeutic effect^a^ Contraindications to use
Definition of adequate trials	Appropriate dose^a^ Appropriate duration^a^	Period of time during which an appropriate dose of medicine is administered, typically at least 2 months at optimal or maximum tolerated dose, unless terminated early due to adverse effects	Not defined	Prophylactic migraine medications in adequate dosages used for at least 3 months each.

*DHE* Dihydroergotamine, *HIT-6* Headache Impact Test, *ICHD* International Classification of Headache Disorders, *MIDAS* Migraine Disability Assessment Test

^a^Not further defined

## Importance of defining refractory migraine

There are numerous reasons to better define and characterize refractory migraine [5–7]. A widely accepted definition of refractory migraine will allow better characterization of the disorder and enable identification of the optimum therapeutic strategy. The epidemiology of refractory migraine in population samples is unknown and the unmet medical need of the patients is largely undefined. In the Refractory Headache Survey conducted by the AHS, the estimated prevalence of refractory migraine in responders’ practice ranged from “less than 5%” to “greater than 31%” (median 5–10%) [7]. It is unknown whether there are differences in the clinical phenotype, genetic makeup, or serum and neuroimaging biomarkers of refractory patients compared to those who are responsive to treatments.

Improved recognition of refractory migraine will help patients obtain the appropriate level of care. The headache characteristics, drug usage, disability status and comorbid features are often used to stage illness and triaging of patients to the proper level of care [8]. This may include a multidisciplinary approach, utilizing behavioural medicine and psychological support. The most effective treatment for refractory migraine, whether there should be various levels of triage, and who should be assigned to what level, remains unclear. Defining and studying this group will enable characterisation of the current patterns of treatments and possibly help identify the best treatment modalities.

It would be useful to identify the risk factors for developing refractory migraine. Migraine is a progressive disorder in some patients and modifiable risk factors for progression include obesity, caffeine, medication, overuse, and sleep problems [9]. Migraineurs with major depression reported physical and sexual abuse in higher frequencies compared with those without depression [10]. Whether these factors are also important in refractory migraine is unclear. There are currently no biological markers that predict migraine progression. Identification of biomarkers for refractory migraine has the potential to stimulate research into disease-modifying agents [11].

Patients with refractory migraine are often excluded from clinical trials, particularly of novel pharmacological approaches. Defining this group of patients could serve as the criteria for inclusion in clinical trials. Conversely, in some device trials, refractoriness is defined as having failed only two different preventative medications [12, 13]. This seems a rather low threshold definition of refractory migraine for an invasive device trial. Interestingly, Goadsby et al criticised the patent foramen ovale trialists for performing a device trial in patients who were only required to fail two preventive treatments, yet some of these authors have themselves subsequently performed invasive device trials using similar inclusion criteria [3, 14]. Better definition of refractory migraine will enable the appropriate patients to be recruited to interventional clinical trials.

## Nomenclature

The terms refractory headache and intractable headache have been used interchangeably to describe headache that is difficult to treat or fails to respond to standard headache treatments. The term “intractable” has the following meanings: unmanageable, uncontrollable, impossible to cope with; difficult, troublesome, demanding, refractory and burdensome. The term “refractory” has the following meanings: unmanageable, recalcitrant, intractable. These terms therefore have definitions that appear to overlap. While it has been acknowledged that establishing a consistent nomenclature is important and therefore using a single term is preferable, there is nonetheless disagreement about which term to use. Some authors have advocated the use of the term “intractable” [3, 5] while others have opted for “refractory” [4, 6].

While both these terms are clearly synonymous, both the AHS and EHF consensus statements have used the term “refractory” and therefore this should be the preferred term hereon in [4, 6].

## Requirements for determining refractoriness

A clear understanding of the pathophysiological mechanisms underlying refractory headache are lacking; therefore, establishing a definition or classification scheme based on mechanism (s) is not currently possible. The diagnosis of refractory headache has therefore been based on headache characteristics, the response to pharmacological and non-pharmacological treatments, and headache-related disability (See Table 1).

### Headache diagnosis

The specific headache type must be ascertained using the International Classification of Headache Disorders (ICHD) criteria before assessing refractoriness to treatment. The ICHD classification criteria are widely accepted and it would be reasonable to expect clinicians and clinical trialists to use the latest iteration of these criteria.

The EHF criteria for refractory migraine [6] are limited to CM while the AHS criteria [4] include both episodic migraine (EM) and CM. While both EM and CM patients can be refractory to treatments, there is a case to be made for keeping these two groups separate. Though EM and CM are part of the spectrum of migraine disorders, they are nonetheless distinct clinical entities. CM is a distinct disorder with clinical, epidemiological, sociodemographic, and comorbidity profiles as well as therapeutic response patterns different from that of EM [15]. Separate criteria need to be developed for refractory EM and refractory CM rather than lumping them together.

Regarding medication overuse headache (MOH), the EHF criteria for refractory migraine require that this entity should be ruled out or be adequately treated before a patient can be classified as refractory [6]. On the other hand, the AHS criteria allow MOH patients to be included but apply a modifier to distinguish patients with and without MOH [4]. MOH can be both the cause and the consequence of the refractoriness hence the reason that the EHF committee elected to exclude MOH. However, a distinction needs to be made between “medication overuse” and “medication overuse headache”. Some patients with CM and medication overuse undergo drug withdrawal, remain abstinent from abortive treatments for prolonged periods of time, remain refractory to preventive treatments and subsequently revert to overusing abortive treatments. Patients who may have MOH should be excluded but patients with medication overuse after exclusion of MOH should be included, albeit that the patients with and without medication overuse need to be studied separately.

### Pharmacological treatment failure

#### Abortive or preventive treatments?

The AHS criteria and Silberstein et al require failure to respond to both abortive and preventive treatments to classify a patient as refractory as one treatment alone would not be considered optimal [4, 5]. However, there are operational difficulties with these criteria, for example, a CM patient with highly disabling daily headaches who responds well to abortive treatments but has failed to respond to numerous preventive treatments would not qualify to be categorised as a refractory patient. In view of this, the EHF criteria are only based on non-responsiveness to preventive treatments. The EHF committee take the view that the key for success in CM is prevention (rather than abortive treatment) and refractoriness is a consequence of prophylactic failure [6].

Refractoriness to abortive and preventive treatments are distinct issues. The mechanism of action of acute and preventive treatments, at least for some agents, are different. Refractoriness to acute treatments may not correlate with refractoriness to preventive treatments. In view of this, separate criteria need to be developed for each without conflating the two into one set of criteria. The main challenge in clinical practice is refractory CM and the primary focus in this group should be on preventive treatments.

#### Which and how many preventive treatments?

In preventive headache treatment, refractoriness is defined as failure to respond or contraindications/ intolerance to proven preventive therapies. Table 1 shows the preventive treatments that are outlined by the various criteria for refractory migraine. Some of the treatments are common to all the criteria (beta-blockers, anticonvulsants, tricyclic antidepressants) while others have listed treatments that have a poor evidence base especially in CM. The only preventive treatments that have a good evidence base for efficacy in CM include topiramate [16], Onabotulinumtoxin A [17] and calcitonin gene related peptide (CGRP) pathway monoclonal antibodies [18]. This poses the challenging issue of whether it is appropriate for refractory CM criteria to require trials of treatments that have a poor evidence base.

While the counsel of perfection would be to only use treatments that have a good evidence base in CM, this will prove to be difficult in practise as Onabotulinumtoxin A and CGRP pathway monoclonal antibodies are difficult to access in some/most healthcare systems. A pragmatic compromise would be to use treatments in CM that have an evidence base for efficacy (2 class I or 2 class II based on American Academy of Neurology Scheme for classification of evidence) in EM [19]. The recent AHS consensus statement on integrating new migraine treatments into clinical practice reviewed the evidence base for treatment of migraine and recommended the use of anticonvulsants, betablockers, tricyclic antidepressants, serotonin-noradrenaline reuptake inhibitors, Onabotulinumtoxin A and CGRP pathway monoclonal antibodies to treat CM [20].

The number of preventive medicine classes necessary to meet the criteria for refractory migraine is another vexing issue. The number of classes of treatments required by the various proposals ranges between two and four. Failure of two preventive treatments, recommended by the AHS criteria, seems to be a rather low threshold definition for refractory migraine. This partly pertains to the fact that the term refractory headache is used in various clinical settings (e.g., primary care vs tertiary specialty care), for diverse interventions (e.g., referral to a specialist; enrolment into prophylactic drug study), and different intensities of the intervention (e.g., hospitalization; enrolment into a device or intracranial surgery trial). The AHS committee seems to have set a rather low threshold to accommodate the use of this term in diverse settings for very differing interventions [4]. Silberstein et al have attempted to provide a graded classification scheme but the operational criteria are cumbersome for clinical practice [5]. It seems inappropriate to outlie a set of criteria that on the one hand prompt primary care physicians to refer patients to secondary care and on the other hand are used for defining patients who might be suitable for invasive headache treatments; these groups are diverse and require different criteria rather than trying to merge into one group. At the other end of the spectrum, requiring failure to all treatments would be unrealistic, particularly since, in the absence of evidence, national practice varies so much.

The threshold for the number of clinical trials should ideally be set by ascertaining the number of trials beyond which there are diminishing returns. However, this issue has not been studied thus far in CM. Until this issue is systematically studied, any threshold for the number of preventive treatments required for defining refractoriness will continue to be arbitrary. The authors view is that the threshold of even three or four preventive treatments is too low and consideration needs to be given to failure to respond to five treatments especially when invasive treatments, such as occipital nerve stimulation, are being considered.

#### Definition of an adequate trial

An adequate trial is defined as a period of time during which an appropriate dose of medicine is administered at an optimal or maximum-tolerated dose, unless terminated early due to adverse effects. Specific criteria for the duration of treatment required for determining failure is not well-defined and varies throughout the literature. The AHS and EHF criteria require two- and 3-month trials, respectively, at an optimum dose. This duration does not include the time taken for the gradual upward titration of the dose. The issue of the adequate duration of trial cannot be settled as no controlled trials that provide for length of treatment have been performed. While longer trials would be preferred by clinicians, patients are often keen to move onto a trial of another drug if there has been no beneficial effect after 2 months at an optimum dose.

#### Definition of failed trial of preventive treatment

What constitutes a failed trial of a preventive treatment? The current criteria for a failed trial are vague or simply not defined. An operational definition is required. The endpoint used in clinical trials (> 50% reduction in headache days or migraine days) seems too robust for clinical practice. Indeed, in chronic pain, a 30% reduction in pain (frequency and/or severity) often translates into a meaningful improvement in quality of life albeit that even with this level of improvement the patient may still be highly disabled by the headache disorder [21]. However, accepting a threshold of a 30% improvement for a succesful trial runs the risk of being criticised for setting the bar too low.

Patients can fail a trial of a preventive treatment, even when used for a short duration or at a suboptimal dose, if they have intolerable side effects. Some patients have medical contraindications to the use of specific preventive treatments thereby potentially lowering the threshold for meeting the criteria for refractoriness; these contraindicated agents should only counted amongst the “failed trials” if after all other potential preventive treatments that can be used have been tried.

### Non-pharmacological treatment failure

A number of meta-analytic studies have shown that biofeedback, relaxation, and cognitive–behaviour therapy are efficacious for migraine [22]. However, behavioural treatments are less accessible than pharmacological treatment and more variable in their application. In view of this the AHS committe elected to define refractory migraine as failure of response to pharmacological rather than non-pharmacological treatments [4]. The EHF committee, on the other hand, require adequate treatment of psychiatric or other comorbities by a multidisciplinary team, if available, but do not provide any operational criteria [6].

While trigger, behavioural and nonpharmacological management of patients is a staple of good clinical practice, incorporating all of these variables into a classification scheme, intended for clinical practice interventions or clinical trial eligibility would be complex, difficult to use, overly cumbersome, and bordering on prohibitive [5].

### Headache-related disability

The role of disability in defining and classifying refractory headaches has not been clearly established. The term refractory headache by itself does not infer or reflect disability. If a headache is frequent and untreatable, but has no disabling impact on the patient, it may be appropriate to do nothing, but it still is considered as refractory [5]. Both the AHS and EHF criteria have not included headache-related disability in the criterion for refractoriness, though the AHS criteria include disability measured using MIDAS (Migraine Disability Assessment Test) as a modifier.

While it would clearly be imperative to only recruit patients with severe headache-related disability (as measured by well validated headache-related disability scores e.g. headache impact test-6 [HIT-6] and Migraine Physical Function Impact Diary [MPFID]) into invasive trials, these measures should not be used to define refractory headache [23, 24].

## Refractory chronic migraine criteria: a personal perspective

Any criteria proposed for defining refractory CM needs to be operational otherwise they are open to varying interpretations. The authors recommendations for defining it are outlined in Table 2. Patients need to satisfy the ICHD-III classification criteria for CM and MOH need to be excluded. However, patients who are currently overusing abortive medications but have previously failed to benefit from withdrawal of medications (i.e. have medication overuse but not medication overuse headache) can be included. Patients need to fail five classes of preventive treatments including two of the three agents/classes that have a good evidence base for efficacy in CM (topiramate, Onabotulinumtoxin A, CGRP pathway monoclonal antibodies), provided they are available in the local healthcare system. There are several migraine preventive treatments in development [25]; the proposed criteria will allow inclusion of these treatments as and when there is a good evidence base for their use. An adequate trial needs to be performed for at least 2 months at the optimum dose (excluding the time taken to titrate the dose) unless terminated early due to side effects. Failure to respond to a drug is defined by less than 50% reduction in frequency and/or severity of monthly migraine days, intolerable side effects or contraindication to use.

**Table 2:** Proposed criteria for refractory chronic migraine

Criteria	Definition
A. Primary Diagnosis	1. ICHD-III chronic migraine 2. Medication overuse headache excluded^a^
B. Refractory	Failure to respond to 5 classes of preventive treatments (including 2 from 1 to 3^b^): 1. Topiramate 2. Minimum of two quarterly injections of Onabotulinumtoxin A 3. CGRP pathway monoclonal antibody 4. Betablockers (Propranolol, Metoprolol, Timolol) 5. Tricyclic antidepressant (Amitriptyline) 6. SNRI (Venlafaxine) 7. Sodium valproate/Divalproex sodium 8. Other pharmacological preventive treatments with established efficacy in migraine^c^
C. Adequate Trial	At least 2 month trial at an optimum or maximum tolerated dose (excluding the time taken for the titration o the dose), unless terminated early due to side effects^d^
D. Failed Trial	1. Failure to respond to drug (< 50% reduction in frequency and/or severity of monthly migraine days) 2. Intolerable side effects 3. Contraindication to use

*CGRP* calcitonin gene related peptide, *ICHD* International Classification of Headache Disorders, *SNRI* Serotonin-noradrenaline reuptake inhibitor

^a^Patients who overuse abortive treatments can be included provided medication overuse headache has been excluded

^b^Applicable if available in the local healthcare system

^c^2 class I or 2 class II based on American Academy of Neurology Scheme for classification of evidence [19]

^d^Optimum dose defined as that used in the controlled trials demonstrating efficacy or as outlined by local treatment guidelines

## Epidemiology

While refractory migraine patients are commonly seen in headache specialty clinics, the epidemiology of this subtype of migraine is poorly studied. The only published study reported on 370 consecutive patients attending a tertiary referral headache clinic [26]. Nineteen patients (5.1%) fulfilled the AHS criteria for refractory migraine. The mean age was 43 years and 58% were female. Seventy-nine percent had refractory CM and 21% had refractory EM. Thirty-six percent had MOH.

## Pathophysiology

Migraine is a multiphasic complex disorder that involves multiple pathways and several neurotransmitter systems. The interested reader is referred to some excellent reviews on the pathophysiology of migraine [27–29]. The pathophysiological basis of refractoriness in migraine is unknown though may include impaired modulation and hyperexcitability resulting in upregulation of pronociceptive systems, structural changes and genetic heterogeneity.

Upregulation of pronociceptive systems may rendering some migraine sufferers’ refractory to standard pharmacotherapy, especially in the setting of acute medication overuse. Peripheral and central sensitization occur during migraine attacks [30]. Moreover, when examined during a pain free state, some patients with CM exhibit cutaneous allodynia and lowered thermal and mechanical pain thresholds indicating the potential for activity independent sensitization to occur in some migraine sufferers. The mechanisms involved in central sensitization may include the release of glutamate, substance P, and CGRP from primary afferent neurons, glutamate activation of N-methyl-D-aspartate receptors (NMDA), and activation of glial cells [31]. The upregulation o the pronociceptive mechanism may be at such a high level in refractory migraine patients that the currently available treatments are unable to wind down these mechanisms.

Multiple neurotransmitter pathways are involved in the pathophysiology of migraine and the prominence of any one particular pathway may differ substantially between patients. There is evidence of an important role for dopamine, serotonin, glutamate, orexin, nitric oxide, CGRP, and others in the pathogenesis of migraine. It is therefore unlikely that a drug which targets any single receptor type or subtype will provide robust efficacy for all migraine sufferers or prevail as the treatment of choice. Patients with refractory migraine may have prominence of pathways which the existing drugs do not modulate.

Evidence is increasing for functional and structural brain changes that appear to occur with increasing migraine frequency. Key structural differences in cortical thickness in the somatosensory cortex and insula were found in individuals with high migraine attack frequency, indicating the potential for repeated sensory activation during attacks to lead to adaptive changes in regions of the brain that process sensory information and modulate the affective response to pain [32]. Additionally, as migraine frequency increases, stronger activation is seen in regions that facilitate pain and weaker activation is seen in regions that inhibit pain [33]. In a structural imaging study, brain cortical thickness, cortical surface area, and regional volumes were highly accurate in distinguishing individuals with CM from those with EM and nonaffected controls [34]. These functional and structural changes may play a role in rendering some patients refractory to pharmacotherapeutic agents.

A meta-analysis of genome wide association studies involved 59,674 affected individuals and 316,078 controls from 22 studies has recently been reported [35]. Overall, 38 distinct genomic loci were found to be significantly associated with migraine risk. The genes identified are involved in ion channels, glutamatergic neurotransmission, and neuronal and synapse development; these genes could influence the enhanced cortical excitability that is characteristic of migraine. Genes expressed in vascular and smooth muscle tissues were also identified, indicating that vascular homoeostasis could influence the expression of the disease and might be integral to the pathogenesis of migraine, at least in some subgroups with migraine. Genetic heterogeneity is likely to be a major determinant of the heterogeneity of response to pharmacotherapeutic agents.

## Management of Refractory Migraine

There are several reasons why standard headache treatments fail [36–38]. These reasons include incomplete or inaccurate diagnosis, important exacerbating factors and comorbidities have been missed, non-pharmacological treatment has been inadequate, pharmacotherapy has been inadequate, neuromodulation has not been considered and unrealistic expectations by patients. These factors should be systematically considered in the clinical evaluation of patients with refractory migraine.

### Review the diagnosis

The diagnosis can be incomplete or inaccurate. This issue takes three major forms: a secondary headache disorder goes undiagnosed, a primary headache disorder is misdiagnosed, or two or more headache disorders are present and at least one goes unrecognized. When managing patients with treatments-refractory headaches, it is important to re-evaluate the headache phenotype periodically to ensure that the diagnosis is accurate and, when necessary, perform any pertinent investigations to exclude secondary headaches.

### Identify important exacerbating factors and comorbidities

Important exacerbating factors include medication overuse, dietary or lifestyle triggers, hormonal triggers, psychosocial factors, or the use of other medications that trigger headaches (eg, phosphodiesterase inhibitors, nitrates) and may lead to refractoriness. In the search for exacerbating factors, ask about factors the patient may have identified and then probe for common and uncommon exacerbating factors, especially those that are subject to modification or intervention.

In headache subspecialty practices, medication overuse and withdrawal is a common cause of refractoriness [39]. It is therefore important to specifically establish the patient’s pattern of medication use, including both prescription and over-the counter medication. Patients are often embarrassed about medication misuse and fear that the physician will make harsh judgments. It is therefore important to ask about medication use in an open, non-judgmental manner.

Numerous population-based epidemiological and clinic-based research studies have established the higher prevalence of major depression, bipolar disorder, anxiety, panic disorders, and obsessive- compulsive disorder in patients with migraine compared with the general population and to non-migraine headache sufferers [40, 41]. There is emerging evidence to suggest that psychiatric comorbidity contributes both to the progression of headache and to the treatment refractoriness of a considerable number of patients [42]. Depressed patients are less likely to adhere to medication regimens, are more likely to become discouraged with less than robust or timely results, while anxious patients are fearful of side effects which precludes titration to effective dosages or fearful of headache which drives medication overuse [43]. Identifying these psychiatric comorbidities and consulting the expertise necessary to effectively manage these psychiatric disorders are therefore essential to effectively managing patients with refractory migraine.

Sleep and headache are intimately related. Over- or under sleeping may cause headache, and yet, sleep may relieve headache. Common sleep disorders associated with headache include obstructive sleep apnoea (OSA), periodic leg movement disorder, insomnia, hypersomnia, and circadian rhythm disorders [44]. Headache upon awakening is common with OSA. Insomnia, the most common sleep disorder associated with headache, may reflect anxiety. Routinely screen refractory migraine patients for sleep disorders. There are a numerous validated scales in sleep medicine, such as the Pittsburgh Sleep Quality Index (PSQI), that may be used for screening [45].

### Educate the patient about lifestyle factors

The aim is to help the patient identify precipitating or exacerbating factors and to encourage their modification as well as implement a lifestyle that will make patient less prone to migraine. Rather than making a long list of things to avoid, patients should be encouraged to have regular habits. Inform patients that regular sleep, exercise, meals, hydration, work habits and relaxation are likely to be rewarded by a reduction in headache frequency [46]. Patients should be encouraged to limit the intake of caffeine and alcohol. There is no well-controlled evidence that specific diets ameliorate migraine.

### Consider biobehavioural therapies

Biobehavioural therapy, including cognitive behavioural therapy (CBT) and biofeedback, and relaxation therapies have been shown to be effective in the acute and preventive treatment of migraine [47, 48]. Biobehavioural therapies may be used alone or in conjunction with pharmacologic and interventional treatments. Evidence suggests that combining biobehavioural interventions with pharmacotherapy provides greater benefits than either modality alone [49].

### Optimise pharmacotherapy

The choice of abortive and preventive treatment agents should be based on evidence based guidelines [20, 50]. Systematically trial and optimize the abortive and preventive treatments. These treatments options are outlined in Table 3.

**Table 3:** Treatment options in the management of refractory migraine

	Oral/Nasal	Injectable	Neurostimulation
Acute	• Oral and Intranasal Triptans • High dose NSAIDS • Paracetamol • Antiemetics	• Subcutaneous sumatriptan	• Transcranial magnetic stimulation • External trigeminal nerve stimulation (Cefaly) • Vagal nerve stimulation
Preventive	• Beta-blockers: Propranolol, Metoprolol, Timolol, Atenolol, Nadolol • Anticonvulsants: Topiramate, Valproate • Tricyclics: Amitriptyline • SNRI: Venlafaxine • Angiotensin pathway blockers: Lisinopril, Candesartan • Calcium channel blockers: Flunarizine • Nutraceuticals: Riboflavin, Coenzyme Q10, Magnesium, Feverfew	• Onabotulinumtoxin A • CGRP-pathway monoclonal antibodies	• External trigeminal nerve stimulation (Cefaly) • Transcranial magnetic stimulation • Occipital nerve stimulation • High cervical spinal cord stimulation
Transitional	• Corticosteroids	• Greater occipital nerve block • Multiple cranial nerve blocks • Intravenous dihydroergotamine • Intravenous lidocaine	

*NSAIDS* Non-steroidal anti-inflammatory drugs

The primary focus of treatments in refractory migraine is on preventive strategies. The success of preventive therapy rests as much on the strategy employed when initiating and titrating the medication and establishing realistic patient expectations as it does on which drug is actually selected [51]. Patients often report that they have failed to respond to multiple preventive treatments; however, it is commonplace to learn that the medications which were not effective or could not be tolerated were never used appropriately. Hence, resorting to some of the basic principles outlined below can often enhance outcomes [37].

Start the chosen drug at a low dose and increase slowly by weekly dose increments until therapeutic effects develop. Set an initial target dose and advise the patient to stop prior to reaching the target dose if significant benefit emerges or side effects are noted. However, all too often, the target dose is considered the ceiling dose. If intolerable side effects are not present, the dose can continue to be increased until efficacy is acceptable and/or optimal. Give each treatment an adequate trial of at least 2 months at the maximal tolerated dose or minimal effective dose.

A drug may be selected (e.g. antidepressant in a migraineur with depression) or avoided (beta-blocker in a migraineur with asthma) based on the presence of a comorbid or coexistent illness. However, care should be taken not to undertreat a comorbid disorder by trying to treat two different conditions with one drug.

The common side effects and their frequency in controlled studies should be discussed with patients prior to initiating the trial. Patients often select preventive medications based on side effect profiles they most want to avoid. Therefore, patient preference must be considered as they are more likely to be compliant with a medication they helped select. Most side effects are self-limiting and attenuate over time. Patients should be educated to expect and encouraged to tolerate the early side effects that may develop when a new medication is started. In this way, a substantial reduction in the frequency and severity of migraine attacks may be realized before reflexively withdrawing or discontinuing a therapy prematurely.

Set the expectations for success. Success is defined as: a 50% reduction in attack frequency, a significant decrease in attack duration/severity or an improved response to acute medication. Unless educated, some patients understandably interpret the term “prevention” literally and anything less than complete relief of attacks is equated with “failure” of the drug.

While there is a paucity of controlled evidence to support the use of two or more preventive medications for the treatment of migraine, it is a useful and rational technique in patients who are poorly responsive or considered refractory. This is especially true if a “partial” response is seen with one medication. Combining medications with a presumably different mechanism of action may also yield therapeutic results, minimize the dosage of each medication, and therefore, minimize the side effect profile of each.

Given that preventative medications can take several weeks to exert their full effect, patients often wish to quickly control attack frequency, especially if they are having frequent very severe headache. These patients may benefit from a transitional or bridging treatment. These interventions are not suitable for long-term use and so often require concurrent use with traditional preventative agents. A short course of steroids and nerve blocks can be considered, albeit that the evidence base for their use is relatively sparse [52–56].

When outpatient treatment fails and patients have continuing and severe pain and disability, inpatient level treatment interventions may be required. Detoxification (if necessary) can be carried out and aggressive parenteral treatments initiated to break the headache cycle initiated. Treatments such as intravenous dihydroergotamine and intravenous lidocaine can be used in this setting [57]. Attendant medical and psychological issues can be addressed, and pharmacologic and nonpharmacologic maintenance treatment can be optimised.

### Consider non-invasive and invasive neuromodulation

Several noninvasive devices have been developed for the treatment of patients with migraine. These treatments modulate pain mechanisms involved in headache by stimulating the nervous system centrally or peripherally with an electric current or a magnetic field [58]. The devices available include single-pulse transcranial magnetic stimulation for the acute and preventive treatment of migraine, electrical trigeminal nerve stimulation for the acute and preventive treatment of migraine, and noninvasive vagus nerve stimulation for the acute treatment of migraine.

In highly refractory and severely disabled patients who fail to respond to most pharmacotherapeutic agents and non-invasive devices (when available), invasive neurostimulation can be considered. The options include occipital nerve stimulation and high cervical spinal cord stimulation [12, 59, 60].

### Utilise a multidisciplinary approach

The lack of a comprehensive multimodal and multidisciplinary approach underlies the refractoriness of a substantial proportion of migraine sufferers who do not respond to currently available therapies [37]. These patients can require input from psychiatry for diagnosing and managing comorbid psychiatric disorders as well as pain psychologists for cognitive behavioural therapy, biofeedback and relaxation therapy. Input from pain medicine or neurosurgeons may be required for interventional procedures such as nerve blocks and invasive neuromodulation.

## Conclusion

Refractory migraine poses a challenge for both patients and clinicians. The patients experience high levels of disability and impaired quality of life. Clinicians struggle to effectively manage these patients. Succesfully managing these patients requires enlisting multiple modalities of therapies, often within the context of a multidisciplinary team. Establishing operational criteria that are widely accepted is clearly needed to eliminate the current disarray in the literature. The premise of the criteria proposed herein is that the currently published proposals have a relatively low threshold for defining refractory migraine and are not operational. Standardisation of these criteria as well as validation and further refinements through field testing will be essential for further progress in this area. The pathophysiology of refractory migraine is poorly understood; a better understanding of the pathophysiology of this entity is urgent needed so that better treatments can be developed for this patient group.

## Data Availability

Not applicable

## Abbreviations

AHS: American Headache Society
CGRP: Calcitonin Gene Related Peptide
CM: Chronic Migraine
EHF: European Headache Federation
EM: Episodic Migraine
HIT-6: Headache Impact Test-6
ICHD: International Classification of Headache Disorders
MIDAS: Migraine Disability Assessment Test
MOH: Medication Overuse Headache
